# Physical fitness and mental health impact of a sport-for-development intervention in a post-conflict setting: randomised controlled trial nested within an observational study of adolescents in Gulu, Uganda

**DOI:** 10.1186/1471-2458-14-619

**Published:** 2014-06-18

**Authors:** Justin Richards, Charlie Foster, Nick Townsend, Adrian Bauman

**Affiliations:** 1 Nuffield Department of Population Health, British Heart Foundation Health Promotion Research Group, University of Oxford, Rosemary Rue Building, Old Road Campus, Roosevelt Drive, Headington, Oxford OX3 7LF, UK; 2 School of Public Health, K25 - Medical Foundation Building, The University of Sydney, Sydney, NSW 2006, Australia

**Keywords:** Physical activity, Physical fitness, Sport-for-development, Mental health, Adolescence, Low-income country, Post-conflict

## Abstract

**Background:**

Physical inactivity contributes to poor fitness and mental health disorders. This is of concern in post-conflict low-income settings where non-communicable diseases are emerging and there is limited evidence for physical activity interventions. We examined the effects of a sport-for-development programme on adolescent physical fitness and mental health in Gulu, Uganda.

**Methods:**

We conducted a single-blinded RCT nested within an observational study with three unbalanced parallel groups. Participants were able-bodied adolescents aged 11–14 years. The intervention comprised an 11-week voluntary competitive sport-for-development football league. Participants who did not subscribe for the intervention formed a non-registered comparison group. Boys who registered for the sport-for-development programme were randomly allocated to the intervention or wait-listed. The girls programme subscription was insufficient to form a wait-list and all registrants received the intervention. Physical fitness was assessed by cardiorespiratory fitness (multi-stage fitness test), muscular power (standing broad jump) and body composition (BMI-for-age). Mental health was measured using the Acholi Psychosocial Assessment Instrument for local depression-like (DLS) and anxiety-like (ALS) syndromes. All randomisation was computer generated and assessors were masked to group allocation. An intention-to-treat analysis of adjusted effect size (ES) was applied.

**Results:**

There were 1,462 adolescents in the study (intervention: boys = 74, girls = 81; wait-list: boys = 72; comparison: boys = 472, girls = 763). At four months follow-up there was no significant effect on the boys fitness when comparing intervention *vs* wait-listed and intervention *vs* non-registered groups. However, there was a negative effect on DLS when comparing boys intervention *vs* wait-listed (ES = 0.67 [0.33 to 1.00]) and intervention *vs* non-registered (ES = 0.25 [0.00 to 0.49]). Similar results were observed for ALS for boys intervention *vs* wait-listed (ES = 0.63 [0.30 to 0.96]) and intervention *vs* non-registered (ES = 0.26 [0.01 to 0.50]). There was no significant effect on the girls for any outcomes.

**Conclusions:**

The sport-for-development league in this study had no impact on fitness and a negative effect on the mental health of participating boys. From this research, there is no evidence that voluntary competitive sport-for-development interventions improve physical fitness or mental health outcomes in post-conflict settings.

## Background

Non-communicable diseases (NCDs) and mental health disorders are growing problems in low- and middle-income countries (LMICs) [1]. Their prevalence and impact is worsening with urbanisation and it is thought that post-conflict populations may be at particular risk [1,2]. Physical inactivity contributes to poor fitness and has been associated with urbanisation as well as the emergence of these negative health outcomes in sub-Saharan Africa [3].

A systematic review of the health benefits of physical activity and fitness in school-aged children suggested a positive effect on NCD risk factors and mental health [4]. The 86 papers included in this review primarily reported physical activity interventions from peaceful high-income countries. However, participation in sport continues to be advocated in clinical and community settings as a means to reach marginalised and deprived populations throughout the world. Positive rhetoric that promises broad social benefits has driven the rapid expansion of the sport-for-development sector since the UN International Year of Sport and Physical Education in 2005. This has included claims of positive health outcomes in LMICs and post-conflict settings that are mediated by increased physical activity and fitness levels [5]. However, there is a paucity of rigorous evaluation for existing sport-for-development interventions and very limited contextually relevant evidence that supports these assertions [5].

The current study is the first randomised controlled trial evaluating the health impact of a sport-for-development intervention in a post-conflict setting and provides an important step in addressing the void of evidence in this sector. It is conducted in Gulu, which is the biggest urban centre in Northern Uganda. In 2006, Gulu emerged from more than 20 years of war that was characterised by high levels of internal displacement and child abduction. It subsequently underwent relatively rapid socio-economic development. Although health data available for Gulu are limited, it appears that there are: 1) consistent high levels of communicable diseases; 2) increasing incidence of NCDs; 3) persistent high levels of mental health disorders [6-8]. This study tested the hypothesis that a voluntary community-based sport-for-development football league would improve the fitness and mental health of young adolescents in Gulu.

## Methods

### Study design

This randomised controlled trial (RCT) nested within a prospective observational study was designed to enable subject access and tracking in the challenging local context. It comprised a multi-arm study assessing fitness and mental health in three parallel and unbalanced groups. The intervention was contrasted with a randomly selected control group that was wait-listed and a self-selected comparison group that did not register for the intervention.

### Study setting and participants

The study was completed in Gulu, Uganda. At the time, Gulu was a low-income setting recovering from more than 20 years of civil war that ended in 2006. The conflict was characterised by a high prevalence of internal displacement and the abduction of children who were forced to serve as soldiers and “wives” [9,10]. During the post-conflict period Gulu has undergone rapid socio-economic development and urbanisation [11]. Gulu municipality now has approximately 150,000 inhabitants, which makes it the largest city and the primary commercial hub in Northern Uganda. However, the history of the region and persistent limitations in local capacity make Gulu a challenging physical, social and political environment to conduct research. Despite concerns about the quality of locally collected data about disease prevalence, there are clear signs of epidemiological transition in the municipality [6,7].

In this study, the most urbanised population in Gulu was targeted. Out of 33 primary schools in Gulu municipality, pupils from the ten most centrally located were selected for assessment. Measurements were embedded in the physical education schedule to facilitate participant access and tracking. An assessment day was assigned to each of the selected schools in cooperation with the head-teachers. All pupils enrolled in sixth grade at these schools could take part (boys: n = 873; girls: n = 1,058). During the week prior to the designated assessment day all participants were informed of the measurement process and provided with written information to take home to their parents/guardians. Opt-out consent was obtained from all participants and their parents/guardians either verbally or by using a form that was attached to the study information sheet. The baseline assessment was commenced in July 2010 and only the measurements of adolescents eligible for the intervention (i.e. able-bodied, 11–14 years age) were included in the study. In late November 2010, immediately following the intervention, follow-up measurements were completed for the sixth grade pupils in attendance at the same ten selected primary schools. Participation levels in the baseline and follow-up assessments were different for each of the outcome variables.

### Intervention

The sport-for-development intervention was a community-based programme called the Gum Marom Kids League (GMKL) and took place over an eleven week period. The GMKL aimed to use sport as a vehicle to promote physical fitness and mental health as well as achieve peace-building objectives in the community. All intervention activities took place at the two most central sports fields in Gulu municipality. Adolescents aged 11–14 years in Gulu municipality voluntarily attended a registration day for the GMKL in September 2010 (boys: n = 495; girls: n = 167). Of these, 146 boys and 81 girls had completed at least one test during the previous baseline assessments. Team allocation took place one week after registration day. The registered adolescents were either assigned to a team for the first season of the GMKL or informed that they had been wait-listed for the next season. One week after team allocation, the intervention group commenced a nine-week competitive football league. Adolescents who were wait-listed or not registered for the intervention were not directly targeted by any GMKL activities.

The intervention was delivered by six paid staff who selected and trained 32 volunteer adults from the local community to become football and peace-building coaches. They received two weeks of training to develop their coaching skills prior to the season commencing. The coaches were allocated to a GMKL team that was located near their residence. Each coach was provided with equipment to conduct at least one 1.5 hour training session per week. Each weekend the GMKL participants took part in a 40 minute game of football (boys: 11-a side full field; girls: 7-a-side half field) and various peace-building activities. Coaches were encouraged to promote participation and equal game-time for all team members. Points towards the GMKL trophy were awarded to reflect a broad focus on football results (30%), on-field behaviour (25%), peace-building activities (25%) and community service (20%).

### Group allocation and masking

All registrants for the GMKL were sorted in to lists according to gender, age group (U12: 11–12 years, U14: 13–14 years) and location of residence within Gulu municipality (division). The current season of the GMKL had capacity for a total of 30 boys (i.e. two teams of 15 members) and 20 girls (i.e. two teams of 10 members) per age group in each of the four residential divisions in Gulu.

There was oversubscription of boys for the GMKL and this presented an experimental opportunity to embed an RCT within a larger observational study. Boys who registered for the GMKL were randomly allocated at the level of the individual into the intervention group for the current season or wait-list control group for the following season. Allocation of the registered boys on each list was completed using a computer-generated list of random numbers. Since the number of registered boys was approximately double the number of places available in the intervention (n = 240), simple randomisation procedures for each list were used. For the girls, there was no oversubscription for the GMKL and consequently no wait-list group was formed. Therefore, all of the girls who registered for the GMKL and were measured at baseline were included in the intervention group. All adolescents who were measured at baseline and did not subscribe for the GMKL were included in the non-registered comparison group.

Therefore, the study comprised three groups: 1) intervention group - subjects measured at baseline who registered for the GMKL and were randomly allocated to the current season of the intervention; 2) wait-list control group (boys only) - subjects measured at baseline who registered for the GMKL and were randomly allocated to the following season of the intervention; 3) non-registered comparison group - subjects measured at baseline who did not voluntarily register for the intervention. The final allocation ratio (intervention: wait-list: non-registered) for each of the groups was approximately 1:1:6 for the boys and 1:0:9 for the girls.

To avoid selection bias the identity and performance of those who had been measured at baseline was concealed until after group allocation was complete. The principal investigator (PI) was responsible for generating the random group allocation sequence for the boys and this was implemented by the GMKL personnel. All measurements at baseline and follow-up were conducted by an independent local research team comprising five staff who had been trained by the PI. They collected descriptive data for all participants to facilitate identification and tracking throughout the study, but remained blinded to group allocation.

### Outcome measures

Cardiorespiratory fitness was measured using the multi-stage fitness test (MFT), which is a valid and reliable test for estimating adolescent VO_2_ max [12,13]. The MFT was completed in groups of up to 30 subjects using a standardised audio recording and the results were reported as the speed (km/hr) at the highest completed level [14].

Muscle power and strength also contribute to fitness and the standing broad jump (SBJ) has been advocated as a practical, efficient and reliable indicator in youth [13,15]. Each participant was given three attempts and the results were reported as the longest jump (cm) [14].

Measurements of height (cm), weight (kg) and age (years) were also recorded for all study participants as part of the fitness assessment. These were used to calculate BMI-for-age (BFA) and height-for-age (HFA) z-scores based on 2007 normative values using the WHO AnthroPlus software [16]. BFA calculations were repeated at follow-up to provide an indicator of changes in acute nutritional status [17]. All of the fitness tests were conducted on a flat area of ground in each of the ten schools using locally adapted protocols and equipment.

Mental health status was measured using a modified version of the Acholi Psychosocial Assessment Instrument (APAI). The APAI was developed, validated and reliability tested in Gulu to assess four local mental health syndromes known as twotam, kumu, par and malwor [18]. Items that assessed twotam, kumu and par were combined to give a depression-like syndrome (DLS) total. The indicators for malwor were also retained for an anxiety-like syndrome (ALS) score. The tool was delivered as a guided questionnaire to all participants from each class (50–120 subjects) simultaneously. Pilot testing indicated good internal consistency using Cronbach’s alpha for DLS (α = 0.949) and ALS (α = 0.850). The results were reported as cumulative scores for all items in each of these sub-scales.

Instructions for all measurements were delivered in both English and the local language (Luo).

### Sample size calculation

Data collected during pilot testing of the measurement methods for the MFT (boys: n = 28, mean maximum speed = 10.93 km/hr, *sd* = 1.22; girls: n = 29, mean maximum speed = 9.53 km/hr, *sd* = 1.03) were used to estimate the number of participants required to detect a 5% change with 95% confidence and 85% power. It was estimated that each group should have a minimum of 89 boys and 84 girls. Based on the advice of the Gulu Municipality Education Officer this calculation was adjusted for expected school absenteeism and/or pupils opting out of testing (10%), ineligibility for assessment (5%) and loss to follow-up (5%). The GMKL personnel estimated that 25% of boys and 10% of girls in the community who were eligible for the intervention would register. They expected that there would be approximately twice as many boys register for the intervention as places available and that the number of girls who register would be insufficient to create a wait-list control group. Therefore, after adjusting for the expected uneven group sizes and reviewing school records, we calculated it was necessary to include the ten most centrally located schools to reach a total sixth grade enrolment of 880 boys and 621 girls.

### Statistical analysis

The data were cleaned and checked for outliers. The sample proportions were tabulated according to location of residence, school and history of abduction after being stratified by intervention group and gender. The overall means and standard deviations for each outcome variable at baseline were also calculated. The baseline fitness results were stratified by age and compared to global norms, but there were no appropriate data to enable similar comparisons for mental health [11,13,16].

All subjects who completed baseline measurements were included in the intention-to-treat analysis for each outcome. Full intervention compliance and fidelity was assumed. For subjects lost to follow-up, we assumed no change from baseline. The crude mean, standard deviation and sample size at baseline and follow-up were tabulated stratified according to intervention group and gender for each outcome variable. These were used to calculate 95% confidence intervals and assess between group differences at baseline for each of the outcome variables. All within-group changes were assessed using a paired *t*-test. The between group differences in mean change and their 95% confidence intervals were compared for the intervention and wait-listed groups using a univariate ANOVA (boys only). Standardised effect sizes (ES) were also calculated using a pooled standard deviation. The analysis was then repeated with adjustments for pre-specified covariates (baseline measures) and factors (location of residence, school, history of abduction). Similar analyses were performed comparing changes in the intervention *vs* non-registered (boys and girls) and wait-listed *vs* non-registered groups (boys only). The results for all of the within-group and between-group analyses were tabulated and the threshold for statistical significance was taken as p < 0.05.

Retrospective analyses comparing the mean and 95% confidence intervals of the subjects lost to follow-up to those retained in the study were completed for each outcome variable. The between-group difference and effect sizes described above were subsequently assessed using a per-protocol analysis and compared to the intention-to-treat results.

### Ethical approval

This study was approved by the Oxford Tropical Research Ethics Committee (OXTREC 18–10) and the Ethics Review Committee of Gulu University (GU/IRC/01/6/10). Approval to access schools and conduct testing was granted by the Gulu District Education Officer, Municipality Education Officer, District Sports Officer and the Head Teachers of the target schools.

### Trial registration

The study is registered with Current Controlled Trials. Full details of the study protocol are described at: http://www.publichealth.ox.ac.uk/bhfhprg/staff/academic/justin-richards/study-protocol-dphil-justin-richards.pdf[19].

## Results

### Recruitment and participant flow

**Figure 1:**
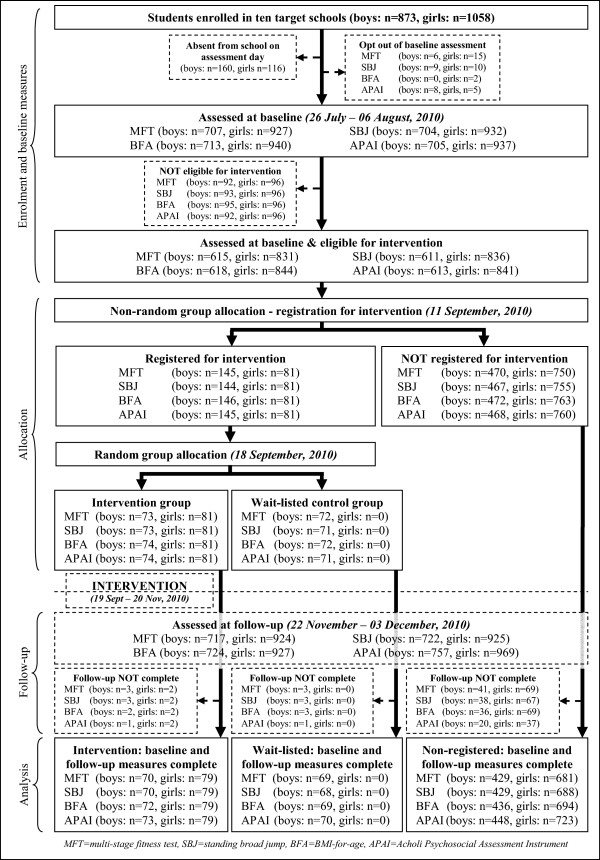
Flow of participants through the study.

There were 160 boys and 116 girls absent from school on the day of testing. The number of adolescents who opted-out of baseline measurement or were ineligible for the intervention varied according to gender and outcome. A total of 618 boys (MFT: n = 615, SBJ: n = 611, BFA: n = 618, APAI: n = 613) and 844 girls (MFT: n = 831, SBJ: n = 836, BFA: n = 844, APAI: n = 841) undertook at least one test at baseline. More students were present at school and agreed to testing at follow-up, but only those who completed baseline measurements were include in the analyses (Figure 1). The overall proportion of participants lost to follow-up was low, but varied according to outcome variable and was higher for those non-registered (boys: intervention = 1.4 - 4.1%, wait-list = 1.4 - 4.2%, non-registered = 4.3 - 8.7%; girls: intervention = 2.5%, non-registered = 4.9 - 9.2%).

### Baseline characteristics

Boys and girls registration rates for the GMKL varied according to school, location of residence within Gulu (division) and history of abduction. Despite randomisation, there were also differences in the demographics of the boys intervention and wait-listed groups that were most notable for location of residence (Additional file 1: Table S1). The baseline performance of all groups in the study was significantly lower for the MFT and significantly higher for the SBJ when compared to global norms established by previous meta-analysis (Table 1) [14,20]. Although the mean BFA and HFA scores were below global means, more than 90% of the sample were in the healthy range (Table 2) [16].

**Table 1:** Comparison of cardiorespiratory fitness and jumping distance of Gulu sample to global normative values

**Gender**	**Age: 11 years**		**Age: 12 years**		**Age: 13 years**		**Age: 14 years**	
	**Gulu**	**Norms**	**Gulu**	**Norms**	**Gulu**	**Norms**	**Gulu**	**Norms**
**Multi-stage fitness test (km/hr): crude mean (95% CI)**								
**Boys**	10.82	10.72	10.69*	10.95	10.95*	11.17	11.32*	11.52
	(10.51 to 11.13)	(10.70 to 10.74)	(10.53 to 10.85)	(10.94 to 10.97)	(10.82 to 11.07)	(11.15 to 11.18)	(11.18 to 11.46)	(11.50 to 11.53)
	n = 44	n = 15 480	n = 141	n = 24 544	n = 232	n = 27 535	n = 198	n = 27 106
**Girls**	9.65*	10.14	9.69*	10.24	9.87*	10.22	9.65*	10.31
	(9.47 to 9.83)	(10.12 to 10.15)	(9.55 to 9.82)	(10.23 to 10.25)	(9.76 to 9.98)	(10.21 to 10.23)	(9.51 to 9.78)	(10.30 to 10.32)
	n = 90	n = 15 446	n = 174	n = 24 255	n = 291	n = 27 110	n = 276	n = 24 924
**Standing broad jump (cm): crude mean (95% CI)**								
**Boys**	165.48*	154.00	171.59*	165.00	182.47*	176.00	195.11*	187.00
	(160.84 to 170.12)	(153.55 to 154.45)	(169.12 to 174.06)	(164.64 to 165.36)	(179.97 to 184.97)	(175.57 to 176.43)	(192.46 to 197.76)	(186.56 to 187.44)
	n = 44	n = 10 045	n = 141	n = 15 313	n = 231	n = 12 052	n = 195	n = 12 415
**Girls**	158.13*	145.00	160.05*	152.00	165.02*	157.00	166.50*	160.00
	(155.00 to 161.26)	(144.57 to 145.43)	(157.71 to 162.39)	(151.65 to 152.35)	(162.92 to 167.11)	(156.59 to 157.41)	(164.37 to 168.63)	(159.59 to 160.41)
	n = 91	n = 9 933	n = 175	n = 16 198	n = 292	n = 12 143	n = 278	n = 12 139

Multi-stage fitness test and standing broad jump global norms sourced from Tomkinson et al. (2008) and Olds et al. (2006) [14,20].

*Statistically significant difference at baseline when comparing means of Gulu sample *vs* normative values (p < 0.05).

**Table 2:** Comparison of body composition of Gulu sample to global normative values

**WHO criteria**	**Age: 11 years**		**Age: 12 years**		**Age: 13 years**		**Age: 14 years**	
	**Boys**	**Girls**	**Boys**	**Girls**	**Boys**	**Girls**	**Boys**	**Girls**
**BMI-for-age: Proportion of sample (%)**								
**Severely thin**	0/44	0/91	4/141	2/177	1/234	0/293	3/199	2/283
	(0.0)	(0.0)	(2.8)	(1.1)	(0.4)	(0.0)	(1.5)	(0.7)
**Thin**	2/44	3/91	11/141	10/177	11/234	8/293	6/199	3/283
	(4.5)	(3.3)	(7.8)	(5.6)	(4.7)	(2.7)	(3.0)	(1.1)
**Healthy**	39/44	85/91	122/141	159/177	213/234	260/293	184/199	246/283
	(88.6)	(93.4)	(86.5)	(89.8)	(91.0)	(88.7)	(92.5)	(86.9)
**Overweight**	3/44	3/91	4/141	6/177	9/234	25/293	6/199	31/283
	(6.8)	(3.3)	(2.8)	(3.4)	(3.8)	(8.5)	(3.0)	(11.0)
**Obese**	0/44	0/91	0/141	0/177	0/234	0/293	0/199	1/283
	(0.0)	(0.0)	(0.0)	(0.0)	(0.0)	(0.0)	(0.0)	(0.4)
**Height-for-age: Proportion of sample (%)**								
**Severely stunted**	0/44	0/91	1/141	1/177	3/234	0/293	3/199	0/283
	(0.0)	(0.0)	(0.7)	(0.6)	(1.3)	(0.0)	(1.5)	(0.0)
**Stunted**	2/44	1/91	3/141	4/177	19/234	2/293	7/199	3/283
	(4.5)	(1.1)	(2.1)	(2.3)	(8.1)	(0.7)	(3.5)	(1.1)
**Healthy**	40/44	89/91	135/141	169/177	212/234	290/293	188/199	275/283
	(90.9)	(97.8)	(95.7)	(95.5)	(90.6)	(99.0)	(94.5)	(97.2)
**>2SD & ≤3SD**	1/44	1/91	2/141	3/177	0/234	1/293	1/199	5/283
	(2.3)	(1.1)	(1.4)	(1.7)	(0.0)	(0.3)	(0.5)	(1.8)
**>3SD**	1/44	0/91	0/141	0/177	0/234	0/293	0/199	0/283
	(2.3)	(0.0)	(0.0)	(0.0)	(0.0)	(0.0)	(0.0)	(0.0)

BMI-for-age and height-for-age categorised according to WHO Guidelines (2007) [16,17].

The girls in the intervention group performed significantly better than the non-registered group at baseline for the MFT (p < 0.001). For the boys, the intervention group scored significantly better than the non-registered group at baseline for the SBJ (p = 0.027) and DLS (p = 0.015). Similar trends were observed for the boys MFT and girls SBJ, but these were not statistically significant. There were no significant differences at baseline between the boys intervention and wait-listed groups for any of the outcome variables (Table 3).

**Table 3:** Baseline measures and follow-up results for all outcome variables

**Gender assessment time**	**Multi-stage fitness test**	**Standing broad jump**	**BMI-for-age**	**Depression-like syndrome**	**Anxiety-like syndrome**
	**(km/hr)**	**(cm)**	**(z-score)**	**(score)**	**(score)**
**Intervention group: crude mean (SD)**					
**Boys**	n = 73	n = 73	n = 74	n = 74	n = 74
**Baseline**	11.14 (0.97)	187.55 (20.85)*	-0.65 (0.80)	21.20 (11.61)*	8.14 (4.50)
**Follow-up**	11.46 (0.96)^#^	181.59 (21.12)^#^	-0.74 (0.84)	24.35 (13.92)	8.73 (4.90)
**Girls**	n = 81	n = 81	n = 81	n = 81	n = 81
**Baseline**	10.10 (0.97)*	166.53 (17.74)	-0.27 (0.86)	31.74 (13.97)	10.13 (4.66)
**Follow-up**	10.43 (0.95)^#^	167.52 (17.39)	-0.32 (0.75)	30.14 (14.16)	9.30 (4.68)
**Wait-listed group: crude mean (SD)**					
**Boys**	n = 72	n = 71	n = 72	n = 71	n = 71
**Baseline**	11.10 (0.93)	184.21 (19.62)	-0.64 (0.85)	24.79 (13.16)	9.01 (5.16)
**Follow-up**	11.58 (0.94)^#^	186.55 (20.52)	-0.71 (0.90)	18.63 (10.32)^#^	7.31 (3.71)^#^
**Non-registered group: crude mean (SD)**					
**Boys**	n = 470	n = 467	n = 472	n = 468	n = 468
**Baseline**	10.96 (1.03)	181.80 (20.58)*	-0.66 (0.90)	24.91 (12.27)*	8.77 (4.48)
**Follow-up**	11.29 (1.08)^#^	181.68 (21.94)	-0.67 (0.89)	22.96 (12.30)^#^	7.87 (4.34)^#^
**Girls**	n = 750	n = 755	n = 763	n = 760	n = 760
**Baseline**	9.70 (0.99)*	163.42 (17.53)	-0.21 (0.90)	32.37 (14.66)	10.35 (4.74)
**Follow-up**	10.19 (1.12)^#^	163.29 (19.31)	-0.25 (0.88)^#^	30.38 (13.64)^#^	9.60 (4.23)^#^

*Statistically significant between-group difference at baseline (p < 0.05).

^#^Statistically significant within-group change (p < 0.05).

### Intervention impact on physical fitness and mental health

Performance in the MFT improved in all groups after the GMKL (boys: intervention p = 0.035, wait-listed p < 0.001, non-registered p < 0.001; girls: intervention p < 0.001, non-registered p < 0.001) (Table 3). However, all of the between-group MFT effect sizes were small and not statistically significant for the crude and adjusted data in both genders. Despite this, there was a noteworthy trend in the results that was consistent across genders. Adjusting the MFT data for baseline, location of residence, school and history of abduction revealed a trend of greater improvement in the intervention and wait-listed boys than those non-registered. The adjusted data for the girls also suggested that the intervention group improved more than the non-registered group (Table 4).

**Table 4:** Intention-to-treat analysis of the effects of the Gum Marom Kids League for all outcome variables

**Gender assessment time**	**Multi-stage fitness test**	**Standing broad jump**	**BMI-for-age**	**Depression-like syndrome**	**Anxiety-like syndrome**
	**(km/hr)**	**(cm)**	**(z-score)**	**(score)**	**(score)**
**Intervention *vs *** **wait-listed: Between group difference in mean change (95% CI) and standardised effect size (95% CI)**					
**Boys (Crude)**					
**Difference**	-0.16 (-0.54 to 0.23)	-8.30 (-14.83 to -1.77)*	-0.02 (-0.20 to 0.16)	9.30 (4.52 to 14.09)*	2.30 (0.61 to 3.99)*
**Effect size**	-0.13 (-0.46 to 0.19)	-0.42 (-0.75 to -0.09)*	-0.04 (-0.36 to 0.29)	0.64 (0.30 to 0.97)*	0.45 (0.12 to 0.78)*
**Boys (Adjust**^**#**^**)**					
**Difference**	0.00 (-0.33 to 0.34)	-4.43 (-10.94 to 2.09)	0.01 (-0.21 to 0.22)	8.18 (4.11 to 12.27)*	2.34 (1.12 to 3.56)*
**Effect size**	0.00 (-0.32 to 0.33)	-0.23 (-0.56 to 0.10)	0.01 (-0.31 to 0.34)	0.67 (0.33 to 1.00)*	0.63 (0.30 to 0.96)*
**Intervention *vs *** **non-registered: Between group difference in mean change (95% CI) and standardised effect size (95% CI)**					
**Boys (Crude)**					
**Difference**	-0.01 (-0.30 to 0.27)	-5.84 (-10.30 to -1.38)*	-0.09 (-0.21 to 0.03)	5.11 (1.84 to 8.37)*	1.49 (0.20 to 2.77)*
**Effect size**	-0.01 (-0.26 to 0.23)	-0.32 (-0.57 to -0.08)*	-0.19 (-0.43 to 0.06)	0.38 (0.14 to 0.63)*	0.28 (0.04 to 0.53)*
**Boys (Adjust**^**#**^**)**					
**Difference**	0.18 (-0.13 to 0.49)	-4.49 (-9.70 to 0.72)	-0.10 (-0.25 to 0.05)	5.16 (1.51 to 8.80)*	1.94 (0.62 to 3.26)*
**Effect size**	0.10 (-0.14 to 0.35)	-0.15 (-0.40 to 0.09)	-0.11 (-0.36 to 0.13)	0.25 (0.00 to 0.49)*	0.26 (0.01 to 0.50)*
**Girls (Crude)**					
**Difference**	-0.16 (-0.11 to 0.44)	1.12 (-2.91 to 5.15)	0.00 (-0.08 to 0.08)	-0.39 (-3.75 to 2.98)	0.08 (-1.14 to 1.30)
**Effect size**	-0.14 (-0.37 to 0.09)	0.06 (-0.17 to 0.29)	0.00 (-0.23 to 0.22)	-0.03 (-0.26 to 0.20)	0.02 (-0.21 to 0.24)
**Girls (Adjust**^**#**^**)**					
**Difference**	0.28 (-0.04 to 0.60)	2.27 (-3.05 to 7.58)	0.02 (-0.09 to 0.12)	-1.61 (-5.72 to 2.51)	0.03 (-1.40 to 1.46)
**Effect size**	0.14 (-0.09 to 0.37)	0.07 (-0.16 to 0.30)	0.02 (-0.21 to 0.25)	-0.06 (-0.29 to 0.17)	0.00 (-0.23 to 0.23)
**Wait-listed *vs *** **non-registered: Between group difference in mean change (95% CI) and standardised effect size (95% CI)**					
**Boys (Crude)**					
**Difference**	0.14 (-0.14 to 0.42)	2.46 (-2.08 to 7.00)	-0.07 (-0.19 to 0.05)	-4.20 (-7.48 to -0.92)*	-0.81 (-2.11 to 0.49)
**Effect size**	0.13 (-0.12 to 0.37)	0.14 (-0.11 to 0.39)	-0.15 (-0.39 to 0.10)	-0.32 (-0.57 to -0.07)*	-0.16 (-0.41 to 0.09)
**Boys (Adjust**^**#**^**)**					
**Difference**	0.18 (-0.12 to 0.48)	1.43 (-3.93 to 6.78)	-0.12 (-0.26 to 0.03)	-3.30 (-6.95 to 0.34)	-0.33 (-1.66 to 0.99)
**Effect size**	0.10 (-0.14 to 0.35)	0.05 (-0.20 to 0.30)	-0.14 (-0.39 to 0.11)	-0.16 (-0.41 to 0.09)	-0.04 (-0.29 to 0.21)

*Statistically significant between-group difference in mean change (p < 0.05).

^#^Data adjusted for baseline, location of residence, school and history of abduction.

The performance of the boys in the intervention group declined significantly for the SBJ (p = 0.011), but there were no significant changes in the wait-listed or non-registered groups. For the girls, SBJ performance remained relatively stable for both the intervention and non-registered groups (Table 3). There were statistically significant crude effect sizes for the SBJ when comparing the boys in the intervention group to those wait-listed (ES = -0.42 [-0.75 to -0.09]) and non-registered (ES = -0.32 [-0.57 to -0.08]), but these were no longer evident in the adjusted analysis. No significant difference was identified between the wait-listed and the non-registered boys when comparing the crude and adjusted data. When comparing the girls in the intervention and non-registered groups, the effect sizes were also small and not statistically significant for both the crude and adjusted SBJ data (Table 4).

The decrease in BFA scores for the intervention and wait-listed groups was not statistically significant. The BFA scores in the non-registered group remained relatively stable for the boys, but there was a statistically significant decrease for the girls (p = 0.001) (Table 3). However, the effect sizes for all between-group comparisons were small and not statistically significant in both genders (Table 4).

The DLS and ALS outcome scores appeared to deteriorate for the boys in the intervention group, but significantly improved in the wait-listed (DLS p < 0.001, ALS p = 0.005) and non-registered (DLS p = 0.001, ALS p < 0.001) groups. The girls in both groups appeared to experience an improvement in their DLS and ALS scores, but this was only statistically significant for those in the non-registered group (DLS p = 0.003, ALS p < 0.001) (Table 3). For the boys, there were significant effect sizes for DLS and ALS when comparing the change in the adjusted data for the intervention group to the wait-listed (DLS: ES = 0.67 [0.33 to 1.00], ALS: ES = 0.63 [0.30 to 0.96]) and non-registered groups (DLS: ES = 0.25 [0.00 to 0.49], ALS: ES = 0.26 [0.01 to 0.50]). The improvement for DLS in the wait-listed boys exceeded that for the non-registered group, but was only statistically significant for the crude data (ES = -0.32 [-0.57 to -0.07]). A similar trend occurred for the boys ALS, but was not statistically significant for the crude or adjusted data. The effect sizes in the girls sample were small and not statistically significant for all between-group comparisons of the DLS and ALS data (Table 4).

There were no other harms or adverse events reported during the study.

### Loss to follow-up

The non-registered adolescents lost to follow-up scored significantly higher at baseline than the rest of their groups for boys SBJ (lost: mean = 190.53 [183.42 to 197.63], retained: mean = 181.03 [179.10 to 182.96]) and girls BFA (lost: mean = 0.05 [-0.14 to 0.23], retained: mean = -0.24 [-0.31 to -0.17]). Although subject retention was very high, the boys lost to follow-up in the wait-listed group also appeared to score highly at baseline for the MFT, DLS and ALS. Conversely, the boy who was lost to follow-up for ALS in the intervention group appeared to score lower than the group average at baseline (Additional file 2: Table S2). The high levels of subject retention in the study meant that per-protocol analyses produced similar crude and adjusted results to those previously reported in the intention-to-treat analyses (Additional file 3: Table S3).

## Discussion

### Principal findings

Contrary to the current evidence for physical activity and mental health, the GMKL intervention adversely affected the depression- and anxiety-like symptoms of the participating boys [4,21]. This occurred despite mental health improvements in the broader community for both genders that were particularly pronounced for the boys in the wait-listed group. There also appeared to be a community-wide increase in cardiorespiratory fitness during this period for both boys and girls. However, the GMKL intervention had no additional effect on the physical fitness of the participants when compared to the wait-listed and non-registered adolescents for both genders.

### Strengths and limitations

This study is the first time a sport-for-development intervention in a post-conflict setting has been independently evaluated using an RCT design. Our results provide an important contribution to demystifying the rhetoric that continues to catalyse mass international investment in the sport-for-development sector. The current paucity of contextually relevant and intervention specific evidence reflects the challenges posed by the limited resources, capacity and security that is typical of post-conflict and low-income settings. We used reliable and valid metrics that assessed local constructs of mental health rather than translating a “western” model of unknown relevance in Gulu [18,22]. The physical fitness measurement methods were also locally adapted, sustainable and reliable. In summary, perhaps the greatest strength of this study was the successful adaptation of rigorous evaluation methods to the practical realities of generating quality evidence in the sport-for-development sector.

However, the challenges of evaluating an independent programme in Northern Uganda tempered the scientific rigour of several components in our study. Participant recruitment was compromised by lower GMKL registration rates and fewer eligible students in sixth grade at the target schools than anticipated. Trial recruitment was further limited by higher levels of school absenteeism than expected at baseline (boys = 18%, girls = 11%). Therefore, the calculated sample size was not realised for the randomised trial and it is possible that the study sample was non-representative for the outcomes of interest. Embedding measurement within physical education classes minimised loss to follow-up, but rates of retention varied among groups. Although there were some differences in baseline measurements for the non-registered adolescents who were lost to follow-up, this was of minimal concern when considering the changes reported in both the intention-to-treat and per-protocol analyses. It was necessary to adjust these analyses for the self-selection bias that occurred when healthier adolescents voluntarily registered for the intervention. Pragmatic constraints precluded longer term follow-up to test if the study outcomes were maintained. This weakened the conclusions that could be drawn about programme effects, which were further limited by the absence of clinically relevant criterion for the metrics utilised [23,24]. The use of locally sustainable emic measurement methods also posed a threat to the external validity of the study findings and limited comparisons to other studies that used different constructs to assess mental health and fitness in similar contexts.

### Interpretation and implications

The principal findings of this study contradict the broad range of social and health-related claims of the sport-for-development sector [25,26]. Our results also challenge the blanket statements that resonate from the physical activity literature suggesting positive mental health outcomes in young people [4,21]. This has implications for programme implementers, policy makers and clinicians who disseminate these messages and promote sport as a physical activity and health intervention.

Perhaps an optimistic interpretation of our mental health results is that the boys directly exposed to the sport-for-development intervention became more comfortable in expressing their problems and this created a response bias. However, the mental health assessment tool used was developed to be sensitive, valid and reliable for local constructs of depression- and anxiety-like syndromes [18,22]. Therefore, it is more likely that there was an inherent component of the GMKL that caused a deterioration in the mental health of the boys directly exposed to the programme. This indicates that the current evidence base for physical activity and mental health is not generically transferrable to sport-for-development interventions. Although several authors have described this gap in the literature and highlighted the rudimentary quality of previous sport-for-development research, the growth of the sector has continued unchecked [5,25-27].

We hypothesise that the adverse impact on mental health of the boys in the GMKL may have been mediated by exposure to new emotional stressors associated with competition. Conversely, the improvement in the mental health of the wait-listed boys beyond that observed in the non-registered group may be attributable to anticipation of participating in the next season of the GMKL. Although the league structure and coaching workshop focussed on community-building initiatives, ethnographic field observations confirm that the majority of the coaches and participants emphasised football performance [28]. These expectations were particularly pronounced in the boys league and may explain the negligible differences in the mental health outcomes observed between the girls groups. A previous evaluation of a sport-for-development intervention in South Africa also indicated that boys focused on winning and associated self-worth with football success [29]. The potential for this to have negative mental health ramifications may be exacerbated in Gulu where the only previous reference point for physical contest was armed conflict. Several authors have emphasised the importance of coaches as “peer leaders” for positive outcomes from sport-for-development interventions and this would appear to be particularly important for interventions where there is only one “winner” [27,30,31]. Process indicators assessing the coach-player interaction were not collected and should be included in future studies to enable consideration of intervention fidelity and coaching quality as components of the programme “dose”.

For the physical fitness outcomes, we hypothesise that a high volume of low-intensity physical activity in the form of active transport (i.e. slow walking to/from school) may have contributed to the large proportion of subjects in the healthy range for BFA at baseline. This may have also contributed to the relatively high performance in the SBJ of the Gulu sample when compared to global norms (i.e. lower body mass to move when jumping). Conversely, the relatively poor performance in the MFT at baseline and the low incidence of malnutrition were consistent with an urbanised setting [1,20]. This suggests low levels of aerobically challenging physical activity and is consistent with the 2003 WHO Global School-Based Student Health Survey that indicated Ugandan adolescents in urban areas engage in low levels of moderate- and high-intensity physical activity [32]. Consequently, there appeared to be potential for improvement in cardiorespiratory fitness and existing guidelines indicate that deconditioned individuals may benefit from introducing training 1–3 days per week [33-35]. Therefore, we postulate that an increase in local capacity to conduct football activities is the most likely explanation for the improvement in MFT seen in all groups. Review level evidence describes the association that physical activity has with cardiorespiratory fitness and mediating environmental determinants such as access to resources [21,36]. Although programme personnel monitored group contamination during GMKL training and matches, it is likely that the equipment provided for the coaches was also used for other activities (e.g. school team training). Despite the non-significant effect sizes, trends in the results suggest that the adolescents most interested in playing football (i.e. intervention and wait-listed groups) improved their MFT performance more than those who did not register for the GMKL (i.e. non-registered group). This supports the hypothesis of differential community-wide exposure to physical activity that was dependant on interest in playing football.

Alternative explanations for the physical fitness results include instability in the MFT metric or seasonal fluctuations associated with physical activity and nutrition. However, the MFT has been shown to be a valid and reliable measure and the changes in the adjusted SBJ and BFA scores suggested there was no generalised temporal effect on the fitness for the entire sample [12,13].

## Conclusions

In conclusion, this study supports the notion that sport is only part of a greater social phenomenon that surrounds it when delivered as a mental health intervention [27]. Despite community-wide improvements in cardiorespiratory fitness, only the boys who participated in the competitive sport-for-development programme experienced negative mental health outcomes. It is possible that the concurrent improvement in fitness and mental health in all of the other study groups for both genders may have resulted from increased local capacity and resources for engaging in physical activity. Therefore, the added benefit of a competitive sport-for-development league on top of improving opportunities to engage in recreational physical activity is not clear. This is of particular concern to clinicians and policy makers given the long term detrimental behavioural effects of negative physical activity experiences during adolescence [37,38]. Results from this study do not support the inclusion of competitive leagues in sport-for-development interventions that aim to improve fitness and mental health.

## Summary

### Article focus

– The positive rhetoric that pervades the sport-for-development sector is not supported by any experimental or observational studies that assess physical fitness or mental health outcomes in post-conflict and low-income settings.

– The only published literature review of the health impact of sport-for-development interventions cited physical activity studies conducted in peaceful high-income settings and called for more research in low- and middle-income countries.

– The purpose of this study was to improve the existing evidence for sport-for-development interventions by evaluating the physical fitness and mental health impact of an existing programme in Gulu, Northern Uganda – a post-conflict and low-income setting.

### Key messages

– Contrary to the current evidence for physical activity and mental health, voluntary and competitive sport-for-development leagues in post-conflict contexts may negatively affect adolescent depression- and anxiety-like syndromes.

– Improving the local capacity and resource provision for non-competitive recreational physical activity may be an effective way to promote adolescent fitness and mental health in post-conflict and low-income settings.

– Rigorous evaluation of sport-for-development interventions is indicated to identify effective programme components and to prevent unexpected harms for the participants.

### Strengths and limitations of this study

– This study included the first RCT assessing the health impact of a sport-for-development intervention in a post-conflict setting and used locally adapted reliable and valid measures.

– Assessing programme effect and external validity was limited by the logistical challenges of conducting research in a unique post-conflict and low-income setting. This required the use of emic metrics and hindered subject recruitment, retention and long-term follow-up.

## Abbreviations

ALS: Anxiety-like syndrome; APAI: Acholi psychosocial assessment instrument; BFA: BMI-for-age; DLS: Depression-like syndrome; ES: Effect size; GMKL: Gum Marom Kids League; HFA: Height-for-age; LMIC: Low- and middle-income country; MFT: Multi-stage fitness test; NCD: Non-communicable disease; RCT: Randomised controlled trial; SBJ: Standing broad jump; SFD: Sport-for-development.

## Competing interests

All authors have completed the ICMJE uniform disclosure form at http://www.icmje.org/coi_disclosure.pdf (available on request from the corresponding author) and declare: no support from any organisation for the submitted work; no financial relationships with any organisations that might have an interest in the submitted work in the previous three years; no other relationships or activities that could appear to have influenced the submitted work.

## Authors’ contributions

JR was the chief investigator and led all aspects of the research (i.e. literature search, figures, study design, data collection, data analysis, data interpretation, writing) as part of DPhil in Public Health at the University of Oxford. CF provided supervisory guidance for all aspects of the research and editorial input for drafts of this manuscript. NT and AB provided editorial input for analysis and for the drafts of this manuscript. JR is the guarantor. All authors read and approved the final manuscript.

## Pre-publication history

The pre-publication history for this paper can be accessed here:

http://www.biomedcentral.com/1471-2458/14/619/prepub

## Supplementary Material

Additional file 1: Table S1 Baseline demographic characteristics of study participants. Click here for file

Additional file 2: Table S2 Difference at baseline between completers vs. lost to follow-up. Click here for file

Additional file 3: Table S3 Per-protocol analysis of the effects of the Gum Marom Kids League for all outcome variables. Click here for file
