# Supplementary material for: Physical fitness and mental health impact of a sport-for-development intervention in a post-conflict setting: randomised controlled trial nested within an observational study of adolescents in Gulu, Uganda
Source: BMC Public Health. 2014 Jun 18;14:619. doi: 10.1186/1471-2458-14-619 (PMC4079830; doi:10.1186/1471-2458-14-619)
Supplement: Additional file 1: Table S1 — Baseline demographic characteristics of study participants. [file 1471-2458-14-619-S1.doc]

| ***Table S1*: Baseline demographic characteristics of study participants** | | | | | | | | | | | | | | | | |
| --- | --- | --- | --- | --- | --- | --- | --- | --- | --- | --- | --- | --- | --- | --- | --- | --- |
| **Factors** | **Intervention** | | |  | **Wait-list control** | | | **Non-register comparison** | | | | | **Total** | | | |
| **(location of residence, school, history of abduction)** | Boys | Girls |  | | Boys | Girls | |  | | Boys | Girls |  | | Boys | | Girls |
|  | (n=74) | (n=81) |  | | (n=72) | (n=0) | |  | | (n=472) | (n=763) |  | | (n=618) | | (n=844) |
| **Location of residence: n (% of allocated group)** | | | | | | | | | | | | | | |  | |
| **Bardege** | 7 | 9 |  | | 5 | | - |  | 36 | | 48 |  | | 48 | | 57 |
|  | (9.5) | (11.1) |  | | (6.9) | | - |  | (7.6) | | (6.3) |  | | (7.8) | | (6.8) |
| **Laroo** | 14 | 5 |  | | 8 | | - |  | 101 | | 138 |  | | 123 | | 143 |
|  | (18.9) | (6.2) |  | | (11.1) | | - |  | (21.4) | | (18.1) |  | | (19.9) | | (16.9) |
| **Layibi** | 22 | 30 |  | | 27 | | - |  | 115 | | 178 |  | | 164 | | 208 |
|  | (29.7) | (37.0) |  | | (37.5) | | - |  | (24.4) | | (23.3) |  | | (26.5) | | (24.6) |
| **Pece** | 31 | 37 |  | | 32 | | - |  | 220 | | 399 |  | | 283 | | 436 |
|  | (41.9) | (45.7) |  | | (44.4) | | - |  | (46.6) | | (52.3) |  | | (45.8) | | (57.7) |
| **School attended: n (% of allocated group)** | | | | | | | | | | | | | | | | |
| **Christ Church** | 6 | 2 |  | | 7 | | - |  | 36 | | 58 |  | | 49 | | 60 |
|  | (8.1) | (2.5) |  | | (9.7) | | - |  | (7.6) | | (7.6) |  | | (7.9) | | (7.1) |
| **Gulu Police** | 6 | 4 |  | | 6 | | - |  | 98 | | 150 |  | | 110 | | 154 |
|  | (8.1) | (4.9) |  | | (8.3) | | - |  | (20.8) | | (19.7) |  | | (17.8) | | (18.2) |
| **Gulu Prison** | 13 | 5 |  | | 12 | | - |  | 46 | | 93 |  | | 71 | | 98 |
|  | (17.6) | (6.2) |  | | (16.7) | | - |  | (9.7) | | (12.2) |  | | (11.5) | | (11.6) |
| **Gulu Public** | 14 | 29 |  | | 13 | | - |  | 45 | | 75 |  | | 72 | | 104 |
|  | (18.9) | (35.8) |  | | (18.1) | | - |  | (9.5) | | (9.8) |  | | (11.7) | | (12.3) |
| **Gulu Town** | 2 | 3 |  | | 2 | | - |  | 31 | | 41 |  | | 35 | | 44 |
|  | (2.7) | (3.7) |  | | (2.8) | | - |  | (6.6) | | (5.4) |  | | (5.7) | | (5.2) |
| **Holy Rosary** | 8 | 15 |  | | 9 | | - |  | 62 | | 94 |  | | 79 | | 109 |
|  | (10.8) | (18.5) |  | | (12.5) | | - |  | (13.1) | | (12.3) |  | | (12.8) | | (12.9) |
| **Labor Line** | 9 | 12 |  | | 7 | | - |  | 17 | | 54 |  | | 33 | | 66 |
|  | (12.2) | (14.8) |  | | (9.7) | | - |  | (3.6) | | (7.1) |  | | (5.3) | | (7.8) |
| **Layibi Central** | 8 | 8 |  | | 8 | | - |  | 50 | | 84 |  | | 66 | | 92 |
|  | (10.8) | (9.9) |  | | (11.1) | | - |  | (10.6) | | (11.0) |  | | (10.7) | | (10.9) |
| **Pece P7** | 3 | 3 |  | | 5 | | - |  | 56 | | 79 |  | | 64 | | 82 |
|  | (4.1) | (3.7) |  | | (6.9) | | - |  | (11.9) | | (10.4) |  | | (10.4) | | (9.7) |
| **St Peter's** | 5 | 0 |  | | 3 | | - |  | 31 | | 35 |  | | 39 | | 35 |
|  | (6.8) | (0.0) |  | | (4.2) | | - |  | (6.6) | | (4.6) |  | | (6.3) | | (4.1) |
| **History of abduction: n (% of allocated group)** | | | | | | | | | | | | | | | | |
| **Yes** | 12 | 16 |  | | 13 | | - |  | 62 | | 130 |  | | 87 | | 146 |
|  | (16.2) | (19.8) |  | | (18.1) | | - |  | (13.1) | | (17.0) |  | | (14.1) | | (17.3) |
| **No** | 62 | 65 |  | | 59 | | - |  | 410 | | 633 |  | | 531 | | 698 |
|  | (83.8) | (80.2) |  | | (81.9) | | - |  | (86.9) | | (83.0) |  | | (85.9) | | (82.7) |
| Data are reported for the study participants who completed at least one test during baseline measurement. | | | | | | | | | | | | | | | | |
