# Supplementary material for: Physical fitness and mental health impact of a sport-for-development intervention in a post-conflict setting: randomised controlled trial nested within an observational study of adolescents in Gulu, Uganda
Source: BMC Public Health. 2014 Jun 18;14:619. doi: 10.1186/1471-2458-14-619 (PMC4079830; doi:10.1186/1471-2458-14-619)
Supplement: Additional file 2: Table S2 — Difference at baseline between completers vs. lost to follow-up. [file 1471-2458-14-619-S2.doc]

| ***Table S2*: Difference at baseline between completers *vs.* lost to follow-up** | | | | | | | | |
| --- | --- | --- | --- | --- | --- | --- | --- | --- |
| **Gender** | **Intervention** | |  | **Wait-list control** | |  | **Non-register comparison** | |
|  | **Completers** | **Lost to follow-up** |  | **Completers** | **Lost to follow-up** |  | **Completers** | **Lost to follow-up** |
| **Multi-stage fitness test (km/hr): crude mean (95% CI)** | | | | | | | | |
| **Boys** | 11.17 | 10.50 |  | 11.09 | 11.50 |  | 10.95 | 11.10 |
|  | (10.94 to 11.40) | (8.02 to 12.98) |  | (10.86 to 11.31) | (#) |  | (10.85 to 11.04) | (10.74 to 11.46) |
|  | n=70 | n=3 |  | n=69 | n=3 |  | n=429 | n=41 |
| **Girls** | 10.09 | 10.50 |  | - | - |  | 9.69 | 9.76 |
|  | (9.87 to 10.30) | (-2.21 to 23.21) |  | - | - |  | (9.61 to 9.76) | (9.52 to 10.00) |
|  | n=79 | n=2 |  | - | - |  | n=681 | n=69 |
| **Standing broad jump (cm): crude mean (95% CI)** | | | | | | | | |
| **Boys** | 187.89 | 179.67 |  | 183.78 | 194.00 |  | 181.03 | 190.53 |
|  | (182.83 to 192.94) | (161.36 to 197.98) |  | (178.98 to 188.58) | (163.88 to 224.12) |  | (179.10 to 182.96) | (183.42 to 197.63)* |
|  | n=70 | n=3 |  | n=68 | n=3 |  | n=429 | n=38 |
| **Girls** | 165.82 | 194.50 |  | - | - |  | 163.02 | 167.55 |
|  | (161.97 to 169.68) | (-2.45 to 391.45) |  | - | - |  | (161.72 to 164.32) | (163.09 to 172.01) |
|  | n=79 | n=2 |  | - | - |  | n=688 | n=67 |
| **BMI-for-age (z-score): crude mean (95% CI)** | | | | | | | | |
| **Boys** | -0.66 | -0.12 |  | -0.65 | -0.37 |  | -0.68 | -0.47 |
|  | (-0.85 to -0.47) | (-3.17 to 2.93) |  | (-0.86 to -0.45) | (-2.70 to 1.96) |  | (-0.76 to -0.59) | (-0.80 to -0.14) |
|  | n=72 | n=2 |  | n=69 | n=3 |  | n=436 | n=36 |
| **Girls** | -0.28 | 0.09 |  | - | - |  | -0.24 | 0.05 |
|  | (-0.47 to -0.09) | (-10.58 to 10.76) |  | - | - |  | (-0.31 to -0.17) | (-0.14 to 0.23)* |
|  | n=79 | n=2 |  | - | - |  | n=694 | n=69 |
| **Depression-like syndrome (score): crude mean (95% CI)** | | | | | | | | |
| **Boys** | 21.21 | 21.00 |  | 24.53 | 43.00 |  | 24.80 | 27.55 |
|  | (18.48 to 23.93) | (#) |  | (21.41 to 27.65) | (#) |  | (23.65 to 25.94) | (22.81 to 32.29) |
|  | n=73 | n=1 |  | n=70 | n=1 |  | n=448 | n=20 |
| **Girls** | 31.62 | 62.00 |  | - | - |  | 31.54 | 35.65 |
|  | (28.48 to 34.76) | (11.18 to 112.82) |  | - | - |  | (30.53 to 32.55) | (30.21 to 41.09) |
|  | n=79 | n=2 |  | - | - |  | n=723 | n=37 |
| **Anxiety-like syndrome (score): crude mean (95% CI)** | | | | | | | | |
| **Boys** | 8.19 | 4.00 |  | 8.84 | 21.00 |  | 8.77 | 8.80 |
|  | (7.14 to 9.24) | (#) |  | (7.65 to 10.03) | (#) |  | (8.35 to 9.18) | (6.67 to 10.93) |
|  | n=73 | n=1 |  | n=70 | n=1 |  | n=448 | n=20 |
| **Girls** | 10.11 | 19.50 |  | - | - |  | 10.09 | 10.76 |
|  | (9.09 to 11.13) | (-12.27 to 51.27) |  | - | - |  | (9.76 to 10.43) | (8.94 to 12.57) |
|  | n=79 | n=2 |  | - | - |  | n=723 | n=37 |
| * Statistically significant difference at baseline when comparing means of completers *vs* subjects lost to follow-up (p<0.05).  # No within-group variance (NOTE: this was due to low levels of loss to follow-up). | | | | | | | | |
