# Supplementary material for: Physical fitness and mental health impact of a sport-for-development intervention in a post-conflict setting: randomised controlled trial nested within an observational study of adolescents in Gulu, Uganda
Source: BMC Public Health. 2014 Jun 18;14:619. doi: 10.1186/1471-2458-14-619 (PMC4079830; doi:10.1186/1471-2458-14-619)
Supplement: Additional file 3: Table S3 — Per-protocol analysis of the effects of the Gum Marom Kids League for all outcome variables. [file 1471-2458-14-619-S3.doc]

| ***Table S3*: Per-protocol analysis of the effects of the Gum Marom Kids League for all outcome variables** | | | | | |
| --- | --- | --- | --- | --- | --- |
| **Gender**  **Assessment time** | **Multi-stage**  **fitness test** | **Standing broad**  **jump** | **BMI-for-age** | **Depression-like syndrome** | **Anxiety-like syndrome** |
|  | **(km/hr)** | **(cm)** | **(z-score)** | **(score)** | **(score)** |
| **Intervention *vs* wait-listed: Between group difference in mean change (95% CI) and standardised effect size (95% CI)** | | | | | |
| **Boys (Crude)** |  |  |  |  |  |
| **Difference** | -0.16 (-0.57 to 0.24) | -8.66 (-15.47 to -1.85)* | -0.02 (-0.21 to 0.17) | 9.44 (4.58 to 14.29)* | 2.33 (0.61 to 4.05)* |
| **Effect size** | -0.14 (-0.47 to 0.20) | -0.43 (-0.77 to -0.09)* | -0.04 (-0.37 to 0.29) | 0.64 (0.31 to 0.98)* | 0.45 (0.12 to 0.78)* |
| **Boys (Adjust^#^)** |  |  |  |  |  |
| **Difference** | 0.03 (-0.32 to 0.38) | -5.50 (-12.33 to 1.34) | 0.05 (-0.16 to 0.26) | 9.04 (5.07 to 13.01)* | 2.64 (1.45 to 3.84)* |
| **Effect size** | -0.03 (-0.36 to 0.30) | -0.28 (-0.61 to 0.06) | 0.08 (-0.25 to 0.41) | 0.76 (0.42 to 1.10)* | 0.73 (0.39 to 1.07)* |
| **Intervention *vs* non-registered: Between group difference in mean change (95% CI) and standardised effect size (95% CI)** | | | | | |
| **Boys (Crude)** |  |  |  |  |  |
| **Difference** | -0.03 (-0.34 to 0.27) | -6.08 (-10.84 to -1.33)* | -0.09 (-0.22 to 0.03) | 5.24 (1.88 to 8.59)* | 1.54 (0.21 to 2.86)* |
| **Effect size** | -0.03 (-0.28 to 0.22) | -0.32 (-0.58 to -0.07)* | -0.18 (-0.43 to 0.07) | 0.39 (0.14 to 0.64)* | 0.29 (0.04 to 0.54)* |
| **Boys (Adjust^#^)** |  |  |  |  |  |
| **Difference** | 0.16 (-0.16 to 0.48) | -5.40 (-10.91 to 0.10) | -0.10 (-0.26 to 0.07) | 5.54 (1.80 to 9.28)* | 2.22 (0.87 to 3.56)* |
| **Effect size** | 0.09 (-0.16 to 0.34) | -0.18 (-0.43 to 0.07) | -0.11 (-0.36 to 0.14) | 0.26 (0.01 to 0.51)* | 0.29 (0.04 to 0.54)* |
| **Girls (Crude)** |  |  |  |  |  |
| **Difference** | -0.20 (-0.49 to 0.08) | 1.16 (-3.11 to 5.43) | 0.00 (-0.09 to 0.09) | -0.36 (-3.85 to 3.14) | 0.10 (-1.16 to 1.37) |
| **Effect size** | -0.17 (-0.40 to 0.07) | 0.06 (-0.17 to 0.30) | 0.00 (-0.23 to 0.24) | -0.02 (-0.26 to 0.21) | 0.02 (-0.21 to 0.25) |
| **Girls (Adjust^#^)** |  |  |  |  |  |
| **Difference** | 0.26 (-0.07 to 0.60) | 1.48 (-4.06 to 7.02) | 0.01 (-0.10 to 0.12) | -2.25 (-6.46 to 1.96) | -0.28 (-1.73 to 1.17) |
| **Effect size** | 0.13 (-0.10 to 0.37) | 0.05 (-0.19 to 0.28) | -0.01 (-0.24 to 0.22) | -0.09 (-0.32 to 0.14) | -0.03 (-0.26 to 0.20) |
| **Wait-listed *vs* non-registered: Between group difference in mean change (95% CI) and standardised effect size (95% CI)** | | | | | |
| **Boys (Crude)** |  |  |  |  |  |
| **Difference** | 0.13 (-0.17 to 0.43) | 2.57 (-2.27 to 7.42) | -0.07 (-0.20 to 0.05) | -4.20 (-7.57 to -0.82)* | -0.80 (-2.13 to 0.54) |
| **Effect size** | 0.11 (-0.14 to 0.37) | 0.14 (-0.12 to 0.39) | -0.15 (-0.40 to 0.11) | -0.31 (-0.57 to -0.06)* | -0.15 (-0.40 to 0.10) |
| **Boys (Adjust^#^)** |  |  |  |  |  |
| **Difference** | 0.13 (-0.18 to 0.45) | 1.36 (-4.32 to 7.03) | -0.16 (-0.31 to 0.00) | -3.67 (-7.41 to 0.08) | -0.39 (-1.74 to 0.97) |
| **Effect size** | 0.08 (-0.18 to 0.33) | 0.04 (-0.21 to 0.30) | -0.18 (-0.44 to 0.07) | -0.17 (-0.43 to 0.08) | -0.05 (-0.30 to 0.20) |
| * Statistically significant between-group difference in mean change (p<0.05).  ^#^ Data adjusted for baseline, location of residence, school and history of abduction. | | | | | |
